# IRF-4-Mediated CIITA Transcription Is Blocked by KSHV Encoded LANA to Inhibit MHC II Presentation

**DOI:** 10.1371/journal.ppat.1003751

**Published:** 2013-10-31

**Authors:** Qiliang Cai, Shuvomoy Banerjee, Amanda Cervini, Jie Lu, Andrew D. Hislop, Richard Dzeng, Erle S. Robertson

**Affiliations:** 1 MOE&MOH Key Laboratory of Medical Molecular Virology, School of Basic Medical Sciences, Shanghai Medical College, Fudan University, Shanghai, People's Republic of China; 2 Department of Microbiology and the Tumor Virology Program of Abramson Comprehensive Cancer Center, Perelman School of Medicine at the University of Pennsylvania, Philadelphia, United States of America; 3 School of Cancer Sciences and Medical Research Council Centre for Immune Regulation, The University of Birmingham, Birmingham, United Kingdom; University of Southern California Keck School of Medicine, United States of America

## Abstract

Peptides presentation to T cells by MHC class II molecules is of importance in initiation of immune response to a pathogen. The level of MHC II expression directly influences T lymphocyte activation and is often targeted by various viruses. Kaposi's sarcoma-associated herpesvirus (KSHV) encoded LANA is known to evade MHC class I peptide processing, however, the effect of LANA on MHC class II remains unclear. Here, we report that LANA down-regulates MHC II expression and presentation by inhibiting the transcription of MHC II transactivator (CIITA) promoter pIII and pIV in a dose-dependent manner. Strikingly, although LANA knockdown efficiently disrupts the inhibition of CIITA transcripts from its pIII and pIV promoter region, the expression of HLA-DQβ but no other MHC II molecules was significantly restored. Moreover, we revealed that the presentation of HLA-DQβ enhanced by LANA knockdown did not help LANA-specific CD4+ T cell recognition of PEL cells, and the inhibition of CIITA by LANA is independent of IL-4 or IFN-γ signaling but dependent on the direct interaction of LANA with IRF-4 (an activator of both the pIII and pIV CIITA promoters). This interaction dramatically blocked the DNA-binding ability of IRF-4 on both pIII and pIV promoters. Thus, our data implies that LANA can evade MHC II presentation and suppress CIITA transcription to provide a unique strategy of KSHV escape from immune surveillance by cytotoxic T cells.

## Author Summary

Major histocompatibility complex (MHC) class II is critical for eliciting specific adaptive immune responses against a wide range of pathogenic agents. KSHV as a member of the herpesvirus family has been shown to encode viral proteins for deregulation of the MHC II signaling pathway. In this study, we discovered that a critical viral encoded antigen LANA can significantly reduce MHC II expression by directly targeting CIITA transcription, and that IRF-4 as an activator of the CIITA promoter directly interacts with LANA, which leads to suppression of IRF-4-mediated CIITA expression. Importantly, inhibition of LANA production restores both CIITA and HLA-DQβ, the only one of six MHC II molecules expressed in KSHV-positive PEL cells. This study clearly demonstrates that each MHC II molecule could be precisely deregulated by specific viral antigen to escape from immune surveillance.

## Introduction

Major histocompatibility complex (MHC) class II is known to play critical roles in the induction and regulation of adaptive immune responses to pathogenic agents [1]. In human, there are at least six major MHC II molecules: HLA-DRα, HLA-DRβ, HLA-DPα, HLA-DPβ, HLA-DQα and HLA-DQβ. During the initiation of the immune response, MHC II molecules expressed from antigen presenting cells (APC) are responsible for binding and presenting peptides to CD4^+^ T lymphocytes [2]. This process triggers the activation and proliferation of the T cells and so elicits an immune response directed against the antigen derived from MHC II-bound peptides. All mature B cells constitutively express MHC class II molecules on their cell surfaces and the Class II transactivator CIITA is the master regulator of MHC class II and its downstream gene expression activities. Previous reports showed that genetic mutations of CIITA are tightly associated with pathogenesis linked to Hodgkin lymphoma and primary mediastinal B cell lymphoma [3]. Transfection of CIITA into cell lines and primary cells which normally lack MHC II expression has been shown to be sufficient to induce MHC II expression [4]. Consistent with these studies, MHC II mRNA was barely detectable, and the cell surface expression of MHC II was undetectable in CIITA-deficient cells [5], [6]. In humans, the transcription of CIITA is controlled by a multi-promoter region which harbors 4 independent promoter units [6]. Among these, promoter pI is constitutively activated in dendritic cells, while pIII promoter is designated as the main regulator of CIITA expression in many hematopoietic lineages including B lymphocytes, dendritic cells, monocytes, and activated T cells [7]. Of particular interest to our studies, promoter pIV is predominantly involved in IFN-γ–inducible CIITA expression in APCs as well as other cell types [8]. However, the function of the pII promoter is still poorly understood. For CIITA-mediated MHC II expression by cytokines like IL-4 and IFNγ, it was shown that IFN-γ activates CIITA through the promotion of STAT1 binding to the GAS site, IRF-1/2 to the IRF-E box, and USF-1 to the E-box within the pIV promoter [9]. In contrast, it remains unclear if IL-4 targets the CIITA promoter, although it was previously shown that IL-4 induces MHC II expression [10].

Interferon regulatory factor (IRF)-4 is a member of the IRF family of transcription factors that participates in a variety of immunological events, especially in pathogen recognition, hematopoietic differentiation and immune modulation [11]. IRF-4 is a multi-functional protein containing a conserved DNA-binding domain (binds to consensus or similar DNA sequences like interferon-stimulated response element ISRE and IRF-binding E-box elements) at the N-terminus and a variable portion at C-terminus that defines biological functions [12]. The ability of IRF-4 to serve as both a transcriptional repressor and activator is determined by the DNA context of its specific binding motif and the interactions with specific transcription factors. It is distinct from other IRFs that are known to be involved mostly in regulation of MHC II inducible expression [13]. The expression of IRF-4 is restricted to the lymphoid and myeloid lineage [12], and is involved in regulation of various cellular processes including cytokine signaling and expression [14], proliferation [15], apoptosis [16], DNA repair, metastasis and immune recognition [17]. Deregulation of IRF-4 results in failure to produce antibodies, and impaired functions of some T cells including Th1, Th2, Th9, Th17 and T_reg_ [18]. In addition, emerging evidence shows that IRF-4 activates the CIITA pIII promoter [19], which is involved in the associated pathogenesis linked to EBV-mediated transformation of B lymphocytes, cell growth of multiple myeloma cells [20], [21], as well as acting as a tumor suppressor in early B cell development [22], all of which supports the targeting of IRF-4 by viruses for immune suppression.

Kaposi's sarcoma-associated herpesvirus (KSHV, also called human herpesvirus 8) is a human γ-herpesvirus and a major cause of morbidity and mortality in Kaposi's sarcoma (KS), multicentric Castleman's disease (MCD) and primary effusion lymphoma (PEL) [23], [24], [25], [26]. Like other herpesvirus, KSHV is predominantly latent in most cells of KSHV-associated lesions and only a few viral genes are expressed during latency. The latency associated nuclear antigen (LANA) encoded by open reading frame ORF73, is one of the critical KSHV encoded latent antigens, and is highly expressed in tumor cells of KSHV-associated malignancies [27], [28]. Studies on LANA have demonstrated that it is a multifunctional protein responsible for viral persistence and tumorigenesis through targeting DNA replication, chromosome tethering, anti-apoptosis, cell cycle regulatory, and gene regulatory functions [29], [30], [31], [32], [33], [34], [35]. Increasing evidence suggests that KSHV is equipped with a number of genes designed to counter the host immune response [36], [37], [38]. Although the acidic-repeat region of LANA was demonstrated to act as an inhibitor of MHC class I peptide presentation [39], particularly in the presentation of the ovalbumin-derived H2K^b^-restricted CTL epitope [40], LANA is also able to generate specific peptide for different CD4+ T cells recognition [41]. However, it remains unclear however whether LANA is directly involved in dysregulation of host MHC class II anti-viral immune response.

In this study, we now show that the MHC class II particularly HLD-DQβ, is significantly down-regulated by LANA, and that this inhibition is due to a direct interaction of LANA with IRF-4, which reduces the DNA-binding affinity of IRF-4 with CIITA pIII and pIV promoters in a IL-4- and IFN-γ- independent manner.

## Results

### LANA down-regulates MHC II and CIITA expression

**Figure 1:**
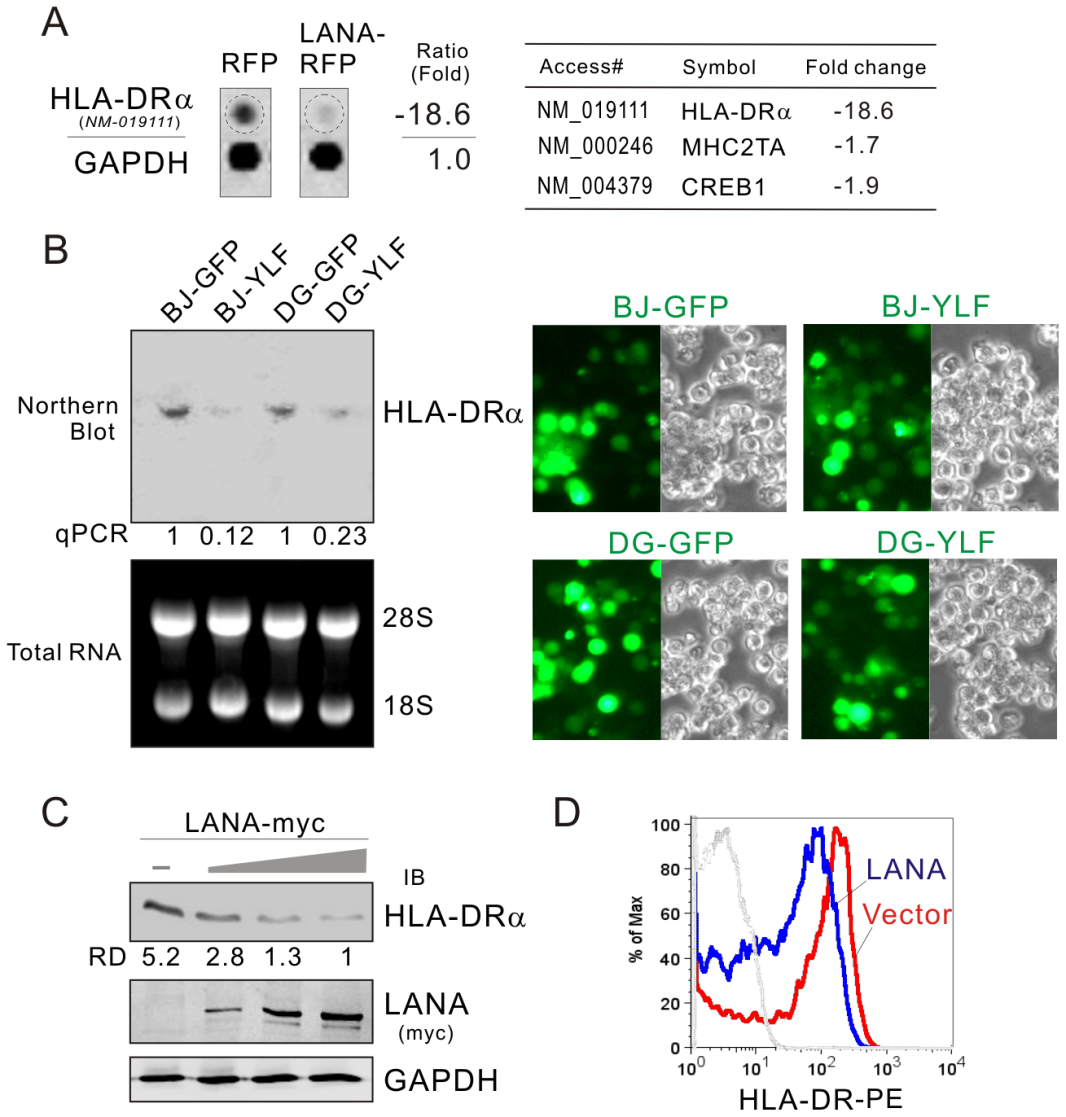
HLA-DRα is down-regulated in LANA-expressing B lymphoma cells. (**A**) The mRNA level of HLA-DRα was reduced in LANA-expressing BJAB cells. The total RNA of BJAB cells with LANA-RFP or vector RFP stable expression were subjected to microarray assay. Representative data and average ratio of HLA-DRα, CIITA and CREB from LANA-RFP and RFP microarray assay are shown. (**B**) Northern analysis of HLA-DRα transcript. The total RNA of BJAB and DG75 cells individually transduced with lentiviruses carrying LANA (YLF) or vector (GFP) alone was subjected to northern blot as described in material and method. The relative density of HLA-DRα transcript is verified by quantitative PCR. The efficiency of lentivirus transduction is shown at the right panels. (**C**) LANA inhibits the expression of HLA-DRα. Ten million of DG75 cells were electroporated with pA3M-LANA (5, 10, 15 µg) or Vector pA3M. At 48-hr post-transfection, the cell lysate were separated by SDS-PAGE and analyzed by Western blot using antibodies HLA-DRα or myc(9E10). Detection of GAPDH was used as an internal control. (**D**) The surface expression of HLA-DR proteins on DG75 cells transfected with LANA-myc or vector was determined by FACS using PE-labeled mAb against HLA-DR.

LANA has been shown to widely interact with several cellular transcription factors. To analyze the overall outcome of these interactions at the level of the cellular gene transcriptome related to immune regulation, the B lymphoma BJAB cells with LANA-RFP stably expressed or RFP vector alone were established and immunology/hematology-related gene mRNA expression profiles were determined by microarray. Microarray analysis revealed that HLA-DRα (a MHC II molecule), CIITA and CREB transcripts were consistently down-regulated more than 18-fold upon LANA expression (Figure 1A). The observed down-regulation of HLA-DRα upon LANA expression was verified by northern blot analysis in both BJAB and DG75 cells transduced with LANA-expressing Lentivirus (YLF) when compared with vector alone control (GFP) at levels ranging from 4–8 fold (Figure 1B). This was further confirmed by the fact that LANA induces a dose-dependent decrease in HLA-DRα expression in DG75 cells with transient transfection up to about 5-fold as expected when compared to the northern blot above (Figure 1C). To determine if HLA-DR presentation was affected by LANA, the levels of HLA-DR at the cell surface in the presence or absence of LANA was determined by flow cytometric analysis. The results showed that LANA dramatically reduced HLA-DRα presentation at the cell surface as seen by the shift in fluorescence peak for LANA-expressing cells (Figure 1D).

**Figure 2:**
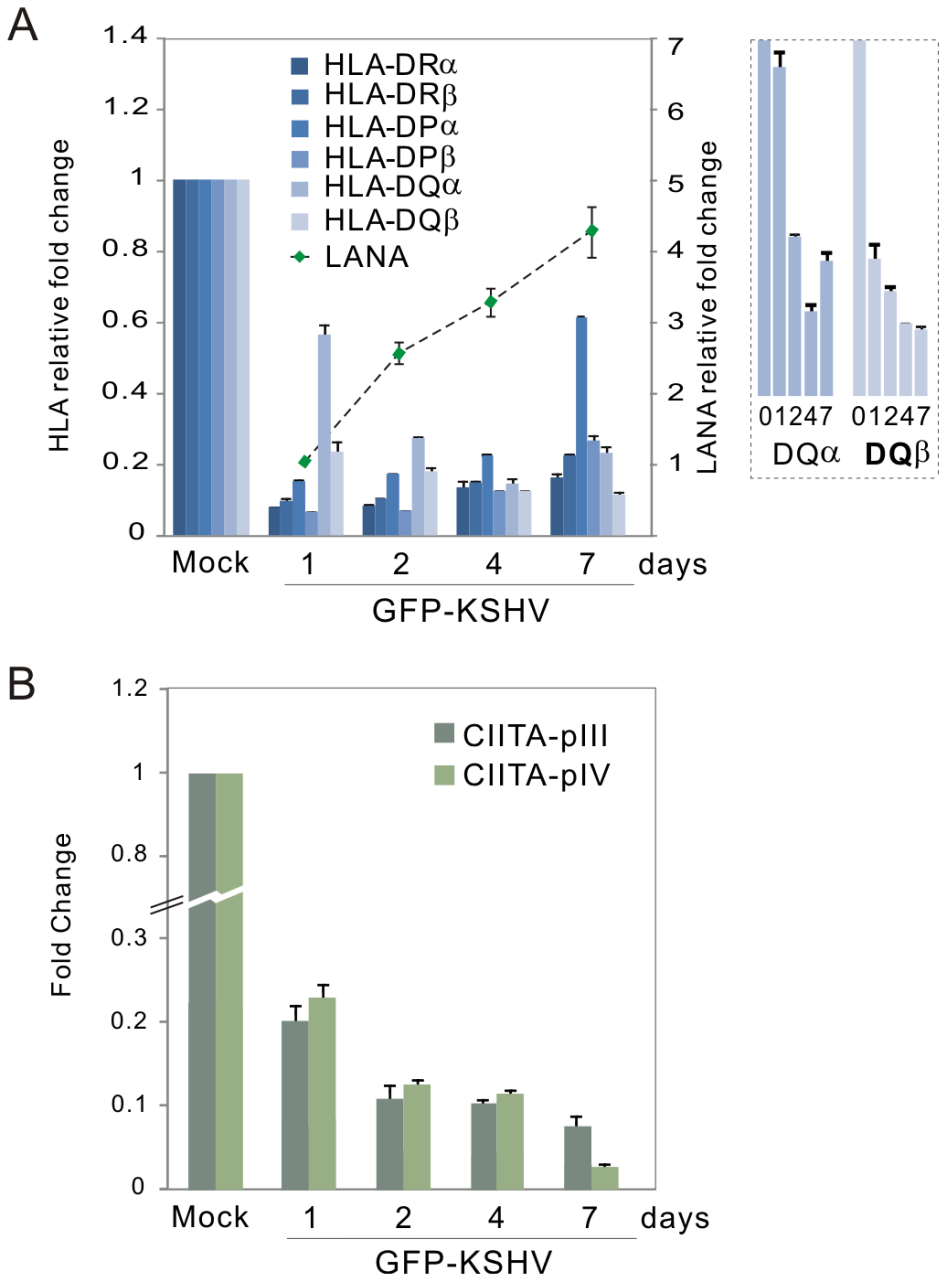
Kinetics of MHC class II expression in PBMCs with KSHV primary infection. Quantitative real-time PCR analysis of (**A**) MHC II and LANA transcripts, and (**B**) CIITA pIII and pIV promoter transcripts, in PBMC with GFP-KSHV infection. The levels of HLA-DQαβ were enlarged on the right panel A. Total RNA was isolated from cells with GFP-KSHV infection for 0, 1, 2, 4 and 7 days. Real-time PCR was performed as described in Materials and Methods. The relative levels of MHCII, LANA and CIITA transcripts were calculated by the cycle threshold (ΔΔCt) values and shown by the fold change compared to mock (day 0) after normalized with GAPDH internal control. All samples were tested in triplicate and the calculation of the mean and standard deviation from two separate experiments. The mock cells without or with GFP-KSHV infection indicated by the relative intensity of LANA were quantified by qPCR shown in the figure.

To determine if other MHCII transcripts and their master regulator CIITA can be down-regulated during KSHV latent infection, de novo infection of human primary cells with GFP-labeled KSHV was performed *in vitro*. Following the standard PBMC infection protocol with GFP-KSHV (Bac36) as described previously [42], we investigated the CIITA pIII and pIV promoter and six subtypes of HLA along with KSHV infection within 7 days. The results of quantitative PCR analysis showed that although the levels of all MHCII transcripts (HLA-DRα, HLA-DRβ, HLA-DPα, HLA-DPβ, HLA-DQα and HLA-DQβ) within 7-day post-infection consistently remained much less than that in the un-infected group (mock), there was a dramatic reduction of both CIITA pIII and pIV promoter-driven mRNA transcripts at 1-day post-infection and gradually decreased up to 7-days post-infection upon LANA expression with a more dramatic reduction seen at the pIV promoter (Figure 2A and B). Interestingly, in addition to the CIITA pIII and pIV transcripts, the reduced level of HLA-DQβ upon KSHV de novo infection was consistent with increased expression of LANA in a dose-dependent manner (Figure 2A, right panel).

**Figure 3:**
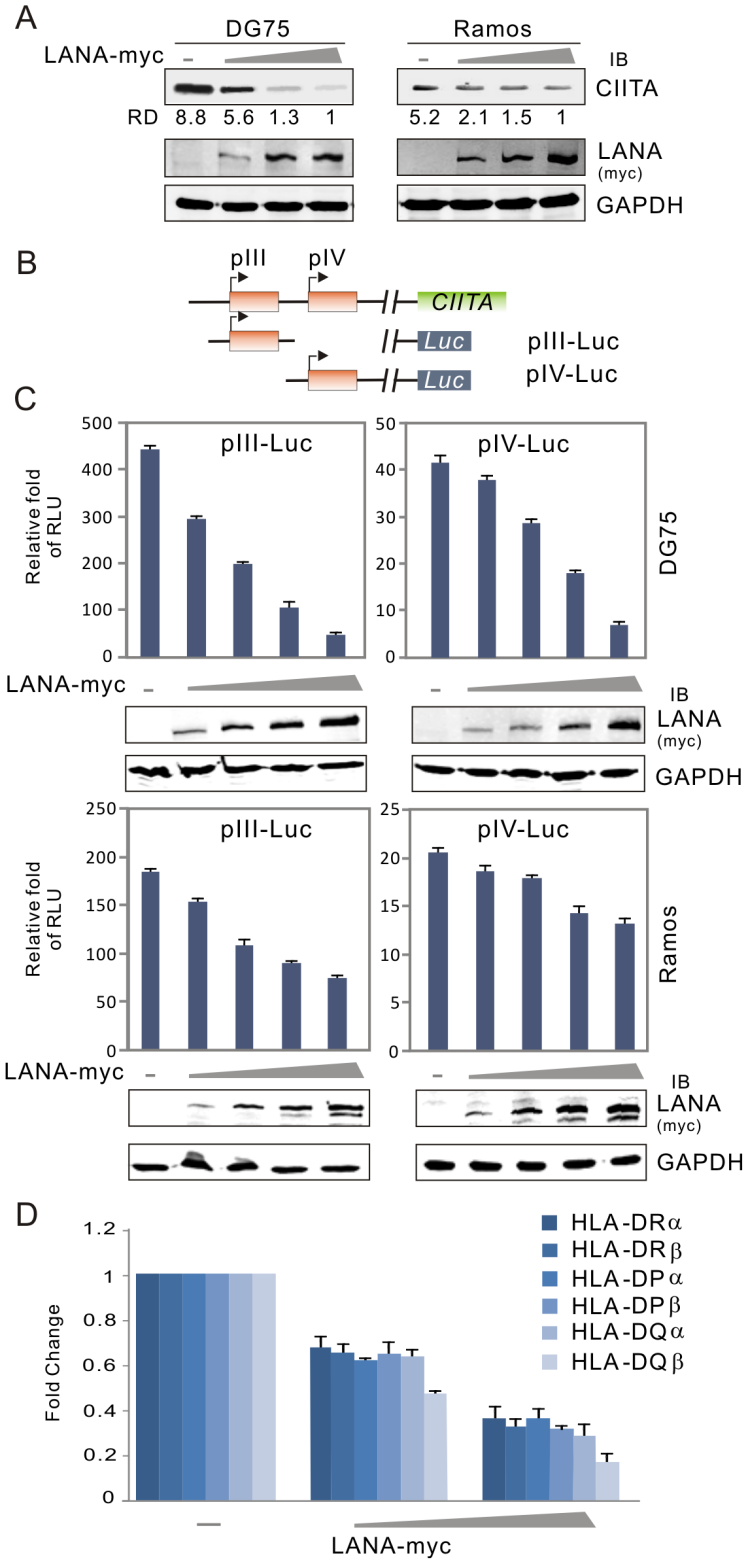
LANA expression represses CIITA expression in B lymphoma cells. (**A**) The level of CIITA expression was reduced by LANA in a dose-dependent manner. Ten million of DG75 or Ramos cells were transfected with a different dose of DNA construct expressing LANA. At 24 hr post-transfection, cells were harvested and lysed for immunoblotting assays as indicated in the figure. GAPDH blot was used as loading control. The relative density (RD) of CIITA proteins was quantified and shown in the figure. (**B**) Schematic representation of CIITA promoter reporters (pIII-Luc and pIV-Luc) with luciferase. (**C**) DG75 and Ramos cells were individually co-transfected CIITA promoter (pIII and pIV) driving luciferase reporter plasmid with either pA3M-LANA or pA3M vector. At 24-hrs post-transfection, cells were harvested and subjected to reporter assay. The results were presented by the RLU (relative luciferase unit) fold compared to pGL3-basic with vector alone. Data is presented as means±SD of three independent experiments. The expression of LANA were detected by immunoblotting and shown at the bottom panels. (**D**) Quantitative real-time PCR analysis of MHCII in DG75 cells transfected with different dose LANA as in panel A.

To confirm if LANA down-regulates CIITA in a dose-dependent manner, we performed luciferase reporter assays by using CIITA pIII or pIV promoter-driven luciferase gene expression, as well as detected endogenous CIITA protein levels by immunoblotting in both DG75 and Ramos B lymphoma cells with an increasing dose of LANA. The results showed that the reduced CIITA protein levels of 5–9 fold were seen with increased LANA expression in the two B-cell line Ramos and DG75 (Figure 3A). Consistently, similar results showing transcriptional inhibition of both pIII and pIV promoters by LANA from the reporter assays in Ramos and DG75 (Figure 3B and C), strongly indicate that down-regulation of CIITA transcripts were tightly associated with LANA expression during KSHV infection. Importantly, the rapid inhibition of MHC II expression during early infection could be critical for KSHV to establish long-term latent infection (which at least is partially contributed by LANA expression, as shown in Figure 3D), we also observed there was a dramatic dose-dependent inhibition of MHC II expression in the presence of LANA alone.

### LANA is essential for inhibition of CIITA and HLA-DQβ expression in PEL cells

**Figure 4:**
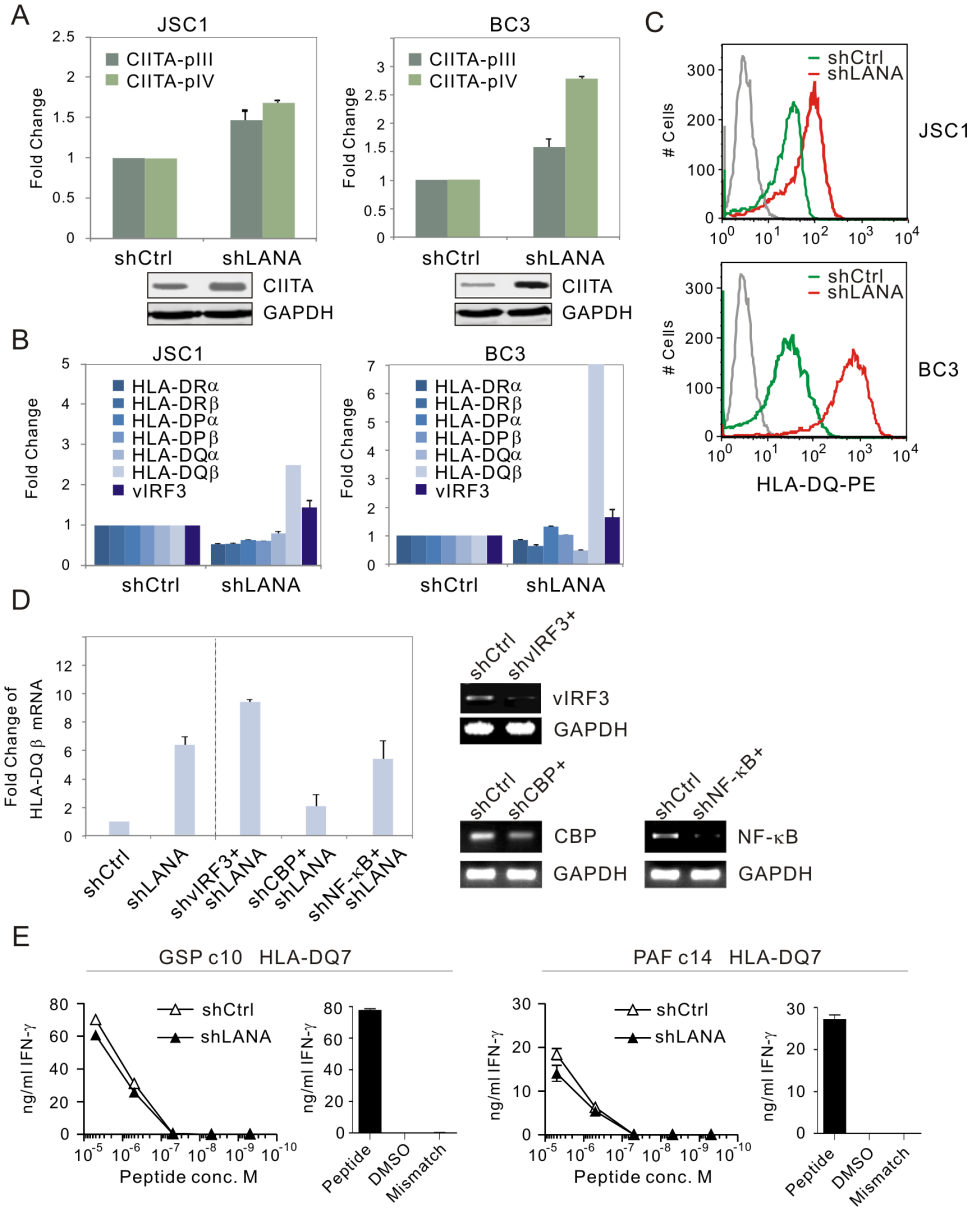
MHCII molecules and CIITA transcript are dramatically enhanced in a LANA-knocked down manner. (**A**) The relative levels of CIITA pIII and pIV transcripts in PEL cells with LANA or control knockdown. Total RNA from PEL (JSC1 and BC3) cells with LANA (shLANA) or control firefly luciferase knockdown (shCtrl) were extracted for RT-PCR analysis. Data from two repeat experiments. The protein levels of CIITA detected by western blotting were shown at the bottom panel. (**B**) The levels of partial MHCII transcripts and vIRF3 were increased in PEL cells with LANA knockdown. (**C**) Cytometric profile of HLA-DQ expression in PEL cells with or without LANA knockdown. PEL cells with LANA or control knockdown were individually divided and subjected to PE-conjugated HLA-DQ antibodies staining. The FACS result is one representative experiment. (**D**) The level of HLA-DQβ transcripts in JSC1 PEL cells with LANA or LANA combined with vIRF3, CBP or NF-κB knockdown. The knockdown efficiency of vIRF3, CBP or NF-κB was presented at right panel by agarose gel after RT-PCR. (**E**) CD4+ T cell recognition of JSC1 primary effusion lymphoma cells transduced with LANA-specific or control shRNA lentivirus. Transduced JSC1 cells were sensitized with ten-fold dilutions of the HLA-DQ7 presented peptides GSPTVFTSGLPAFVS or PAFVSSPTLPVAPIP; peptides are identified in the figure by their first three amino acids. Target cells were incubated in triplicate with cognate epitope-specific CD4+ T cell clones for 18 hours and IFN-γ secreted from these cells into the culture supernatant estimated by ELISA. In parallel T cell clones were assayed against HLA-matched Epstein-Barr virus transformed B cell line LCL sensitized with the peptide or the peptide solvent, or an HLA mismatched B cell line (right panel). Data shown is one representative experiment of two.

To further elucidate the role of LANA on CIITA and MHC II expression, LANA in both KSHV-positive BC3 and JSC1 cell lines was transiently knocked down individually by introduction of small interference RNA specifically against LANA without significantly interrupting or affecting the expression of other latent transcripts [43]. The levels were monitored by quantitative PCR analysis. The results showed that the levels of both CIITA pIII and pIV transcripts were dramatically enhanced once LANA expression was reduced (Figure 4A, the protein level of CIITA was verified by western blotting at the bottom panel). Intriguingly, only HLA-DQβ but no other MHC II molecules (HLA-DRα, HLA-DRβ, HLA-DPα, HLA-DPβ and HLA-DQα), was significantly enhanced upon LANA knockdown in both BC3 and JSC1 cells (BC3 over 2 fold greater compared to JSC1) (Figure 4B). This was further confirmed by using flow cytometric analysis with PE-conjugated antibodies specific against HLA-DQ (Figure 4C). Unexpectedly we also observed that the levels of HLA-DRα, DRβ, and DQα were presented consistently lower after LANA inhibition, while the levels of vIRF3 (a viral antigen previously shown to inhibit MHC II in latent infection [38]) were slightly enhanced (Figure 4B). This supports our hypothesis that LANA not only modulates the transcriptional activity of the CIITA promoter but can directly contribute to regulation of MHC II molecules, in particular HLA-DQβ expression in a CIITA-independent manner, and that LANA can also cooperate with vIRF3 in controlling MHCII expression.

To further determine whether vIRF3 or other LANA-associated transcriptional factors including CBP and NF-κB is involved in LANA-mediated regulation of HLA-DQβ expression, we performed inhibition of vIRF3, CBP or NF-κB with LANA knockdown followed by quantitative PCR analysis of HLA-DQβ transcripts. As shown in the Figure 4D, vIRF3 also inhibited HLA-DQβ expression (although vIRF3 has much less capacity to do so than LANA, ∼1/3 fold), and that CBP but not NF-κB is involved in the up-regulation of HLA-DQβ expression after LANA knockdown.

To determine if up-regulation of HLA-DQ by LANA knockdown will lead to enhanced CD4+ T cell recognition of PEL cells, we assessed their ability to be recognized by LANA-specific T cells. HLA-DQ7-positive JSC1 cells with or without LANA knockdown were sensitized with 10-fold dilutions of the DQ7 presented peptides GSPTVFTSGLPAFVS and PAFVSSPTLPVAPIP[41], before being incubated with the cognate epitope-specific T cell clones, and recognition was assessed by measuring IFN-γ secretion from the T cells. Epstein-Barr virus transformed B cells sensitized with the peptides or peptide solvent were included in parallel recognition assays as controls. We observed that LANA knockdown did not enhance peptide-specific CD4+ T cell recognition of JSC1 (Figure 4E), but did observe a slight decrease in recognition after LANA knockdown (Figure 4E). This suggests that LANA-mediated inhibition of HLA-DQ-peptide presentation may not be LANA itself but due to additional contributions by other antigens.

### IRF-4 is involved in LANA-mediated inhibition of CIITA transcripts

**Figure 5:**
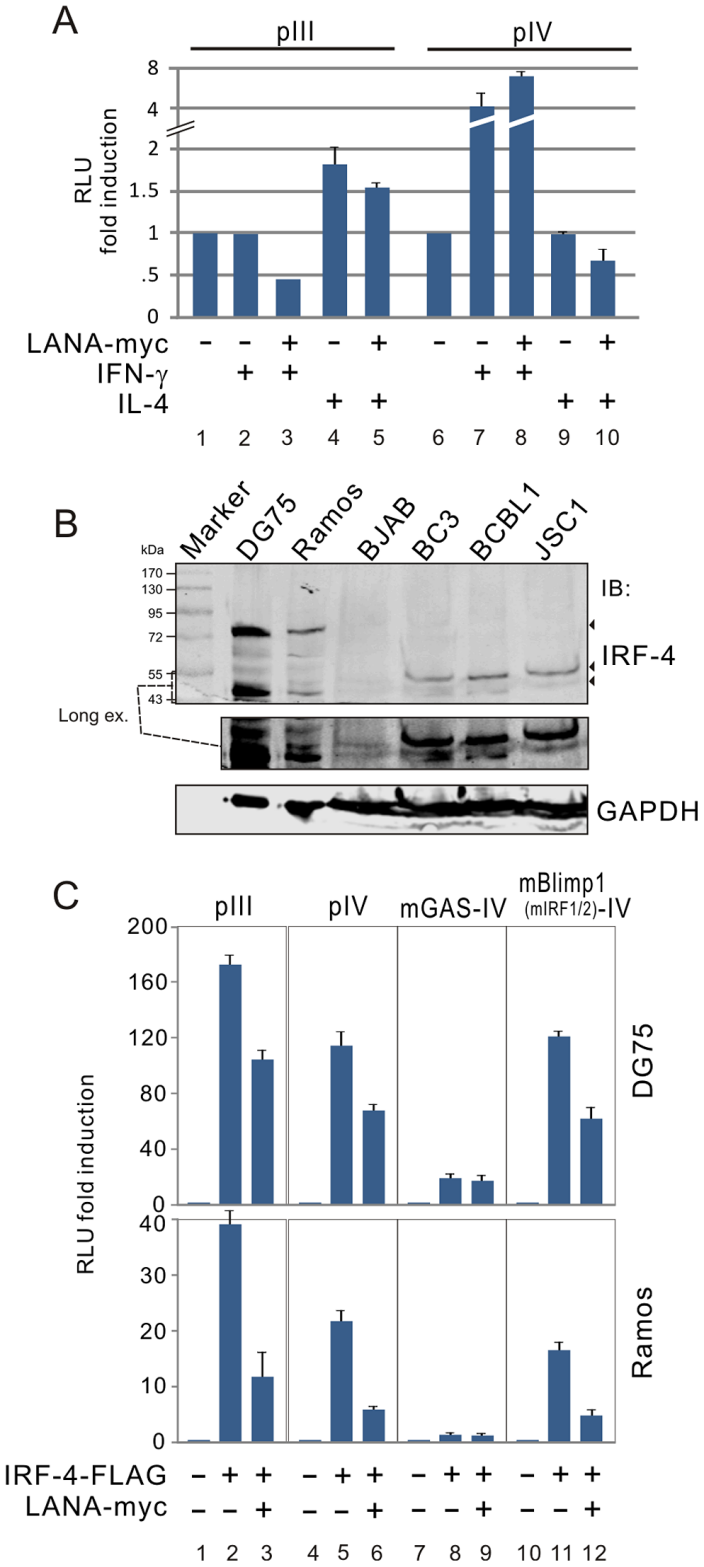
LANA blocks the transcriptional activity of IRF-4 on both pIII and pIV CIITA promoters. (**A**) The inhibition of LANA on CIITA promoter is independent on IL-4 or IFNγ-induced signaling.HEK293 cells were co-transfected pA3M-LANA with pIII or pIV luciferase reporter as indicated in the figure. At 24 hr post-transfection, cells were treated with IL-4 (20 ng/ml), IFNγ (400 ng/ml) or untreated for 36 hours, followed by reporter assays. (**B**) Expression of IRF-4 in KSHV negative B lymphoma and positive PEL cells. Immunoblotting (IB) analysis was performed using 100 µg of whole-cell lysates from KSHV negative B lymphoma (DG75, Ramos and BJAB) and positive PEL (BC3, BCBL1, JSC1) cell lines. Immunoblots were probed with anti-IRF-4 antibody. The membrane was reblotted with anti-GAPDH antibody to verify protein-loading homogeneity. The position of IRF-4 molecular weight around 43–55 kDa with long exposure was presented at the middle panel. (**C**) LANA blocks IRF-4-mediated transcription levels of both pIII and pIV. DG75 and Ramos cells were individually co-transfected CIITA promoter (pIII, pIV, and pIV with GAS or Blimp1(IRF1/2) binding-site mutation) driving luciferase reporter plasmid with different combination of pA3F-IRF-4, pA3M-LANA and vector. At 24 hr post-transfection, cells lysates were subjected to luciferase reporter assays. The results were presented by the RLU (relative luciferase unit) fold compared to each reporter with vector alone. Data is presented as means±SD of three independent experiments.

Previous studies have shown that both IFN-γ and IL-4 can directly or indirectly target different CIITA promoters to regulate MHC II transcription [10], [44], [45], [46], [47]. Given the modulation of CIITA expression by LANA, we performed luciferase reporter assays by using CIITA promoter pIII and pIV reporter in the presence or absence of LANA in both DG75 and HEK-293 cells followed by treatment with IL-4 or IFN-γ. Consistent with previous reports, the results showed that IL-4 and IFN-γ individually induced the transcription levels of the CIITA pIII and pIV promoters respectively regardless of LANA expression in the HEK-293 cells (a similar result was obtained in DG75 cells, data not shown) (Figure 5A, compare lane 1 with lanes 4 and 5; lane 6 with lanes 7 and 8). In contrast, similar inhibitory activity of LANA on both pIII with IFN-γ, and pIV with IL-4 treatment was seen (Figure 5A, compare lanes 2 with 3; lanes 9 with 10). This further suggests that LANA-mediated inhibition of both CIITA pIII and pIV promoters is independent of either IL-4 or IFN-γ stimulation.

To define the potential inhibitory mechanism that LANA has on CIITA promoter pIII and pIV, we examined transcription factor function on these promoters in the presence of LANA. IRF-4 is known to regulate pIII activation [19], and that we and other groups have found that IRF-4 is constitutively expressed in PEL cells (Figure 5B) [48], although an additional IRF-4 protein band located around 72 kDa in both DG75 and Ramos cells while there was much lower expression of IRF-4 in BJAB cells. We asked if IRF-4 was also able to upregulate pIII or pIV promoter activity in the presence of LANA. To do so, we performed similar pIII and pIV promoter-driven luciferase reporter assays in both DG75 and Ramos cells in the presence or absence of IRF-4. Interestingly, we found that similar to the effect of IRF-4 on the pIII promoter (Figure 5C, compare lanes 2 with 1), there was a dramatic enhancement of pIV transcription in the presence of IRF-4 (Figure 5C, compare lanes 5 with 4). Furthermore, in the presence of LANA, the results showed that the effects of IRF-4 on both the CIITA pIII and pIV promoters were dramatically inhibited by LANA (Figure 5C, compare lanes 2 with 3, lanes 5 with 6). This supports our notion that IRF-4 is involved in LANA-mediated inhibition of CIITA transcription.

To define which *cis* element within the CIITA promoter is critical for LANA-mediated inhibition of CIITA transcription through IRF-4, a series of mutants of the CIITA pIV-driven luciferase reporter gene were used [49], and subjected to reporter assays in both DG75 and Ramos cells in the presence or absence of IRF-4 or IRF-4 with LANA. Intriguingly, the activity of CIITA pIV promoter with a specific mutation in the IRF1-binding site (mBlimp1-IV) shows an activity which was the same as that seen for the wild type pIV promoter in the presence of IRF-4 or IRF-4 with LANA (Figure 5C, compare lanes 5 with 11, lanes 6 with 12). However, the pIV promoter with a mutation of STAT1-binding site (mGAS-IV) resulted in a remarkable decrease in the promoter activity in the presence of IRF-4 or IRF-4 plus LANA when compared with wild type pIV (Figure 5C, compare lanes 5 and 6 with 8 and 9). These results strongly suggested that IRF-4 is involved in modulation of both CIITA pIII and pIV by LANA.

### IRF-4 directly interacts with LANA *in vitro* and *in vivo*

**Figure 6:**
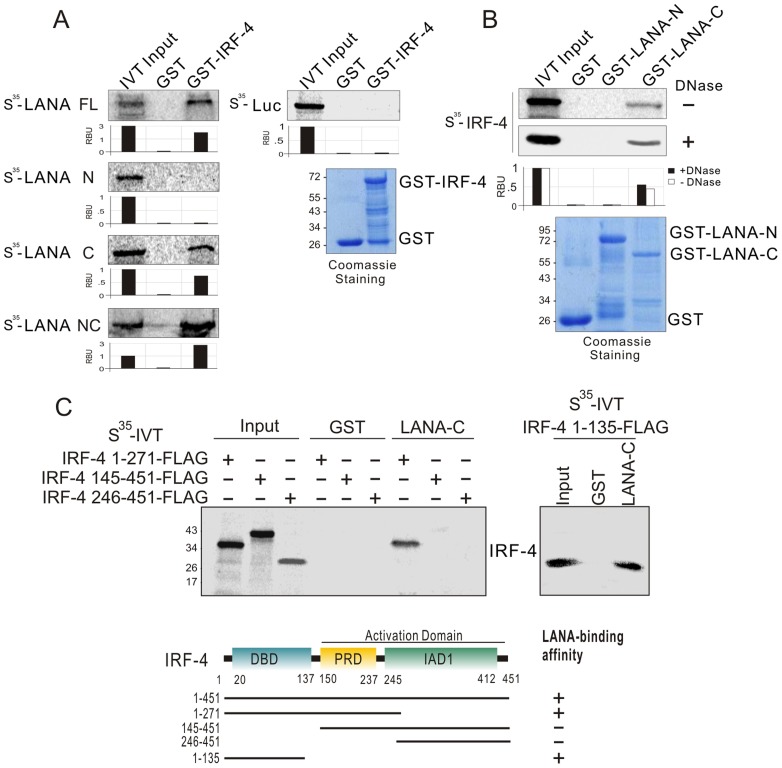
IRF-4 binds with LANA through its DNA-binding domain *in vitro*. (**A**) IRF-4 binds to C-terminal domain of LANA *in vitro*. The ^35^S-radiolabeled *in vitro*-translated proteins of LANA truncated mutants were pre-cleared with GST bead, followed by incubation with GST or GST-IRF-4 beads. The bound protein mixtures were resolved by appropriate SDS-PAGE, and protein species detected by autoradiography. 5% of *in vitro* translated protein is used as input. The quantification of relative amount of bound proteins (RBU) is shown at the bottom. (**B**) The ^35^S-radiolabeled *in vitro*-translated full length IRF-4 was pulled down by truncated mutants of LANA fusion with GST (GST-LANA N1–340 and C945–1162) in the presence or absence of DNase I (20 U/ml) treatment. Coomassie blue staining of purified GST-LANA is shown at the bottom panels. (**C**) LANA binds to N-terminal domain of IRF-4 *in vitro*. The ^35^S-radiolabeled *in vitro*-translated proteins IRF-4 with FLAG tag (1–135, 1–271, 145–451 and 246–451) was pulled down by GST or GST-LANA C. Schematics illustrate different structural domains of IRF-4 with LANA-binding ability.

**Figure 7:**
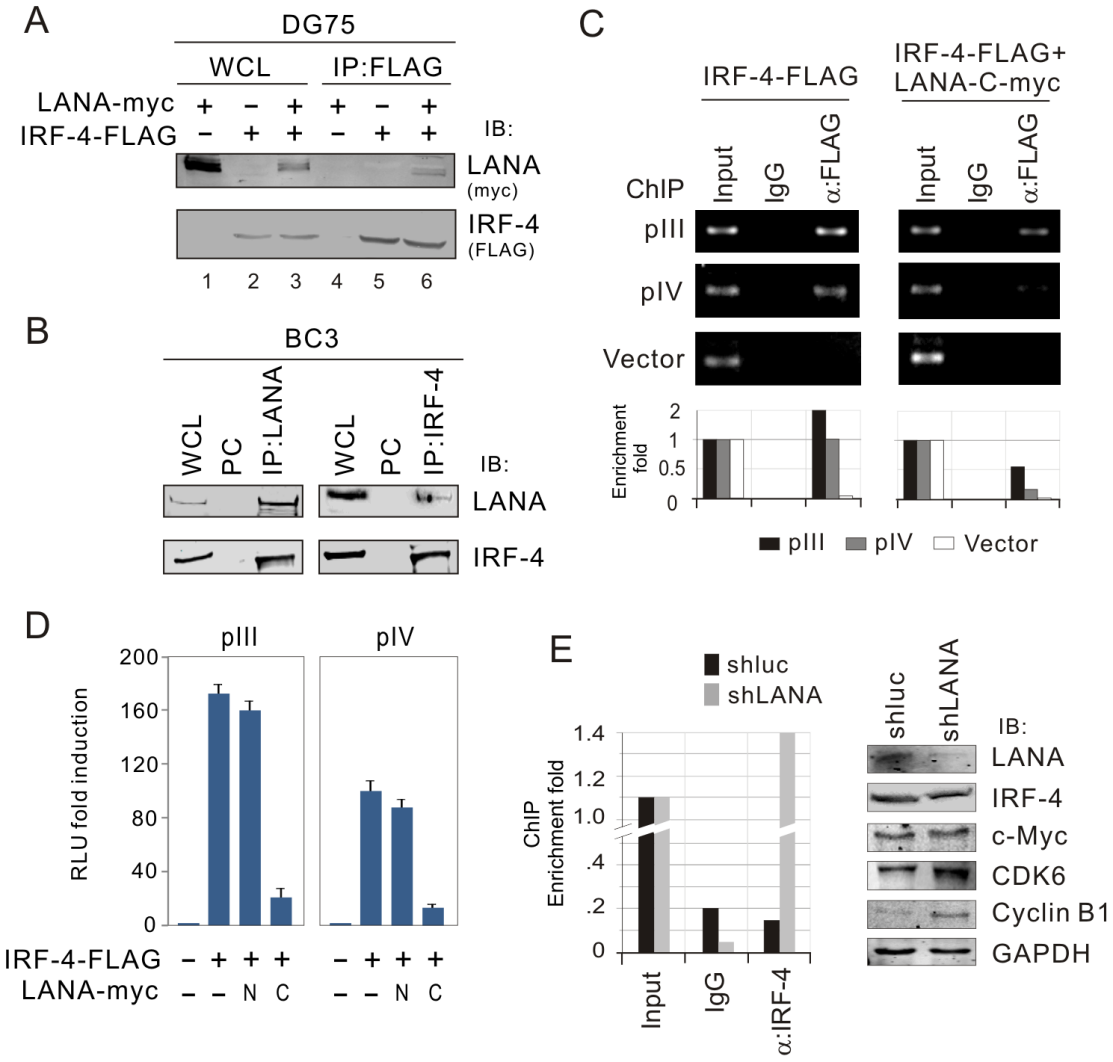
Endogenous IRF-4 interacts with LANA and results in failure of the DNA binding capacity on CIITA promoter. (**A**) Exogenous IRF-4 interacts with LANA. DG75 cells were co-transfected IRF-4-FLAG with pA3M-LANA or vector control. At 48-hr post-transfection, the whole cell lysates (WCL) were subjected to directly immunoblotting (IB) or immunoprecipitation (IP) with anti-FLAG (M2) antibody followed by immunoblotting with indicated antibodies. (**B**) Endogenous IRF-4 associates with LANA. Cell lysates of five million of PEL cells (BC3) were subjected to directly immunoblotting or immunoprecipitation with anti-IRF-4 antibody followed by immunoblotting with indicated antibodies. Normal serum IgG was used for preclear (PC). (**C**) LANA reduces DNA-binding ability of IRF-4 on pIII and pIV promoter. DG75 cells were co-transfected IRF-4-FLAG with or without LANA-C_930–1162_-myc in the presence of pIII, pIV or pGL3-basic vector alone. Chromatin immunoprecipitation assays were performed with anti-FLAG(M2) or normal IgG antibodies control. The DNA-binding ability of IRF-4 was detected by standard (upper) and quantitative real-time (lower) PCR with primers targeting ampicillin gene of pGL3 plasmid. (**D**) The carboxyl terminus of LANA dramatically blocks the transcriptional activity of IRF-4 on both pIII and pIV promoter. Ten million DG75 cells were individually co-transfected with different combination of IRF-4-FLAG, LANA1-340-myc (N), LANA 930–1162(C) or vector alone as indicated in the figure. At 24 hr post-transfection, cells lysates were subjected to luciferase reporter assays. The results were presented by the RLU (relative luciferase unit) fold compared to each reporter with vector alone. Data is presented as means±SD of three independent experiments. (**E**) Knockdown of LANA allows IRF-4 to bind to the CIITA promoter. ChIP analysis of endogenous CIITA pIII promoter in BC3 cells with or without LANA knockdown was conducted using normal mouse IgG, α-IRF-4 agarose, and subjected to qPCR analysis using primers indicated in Figure 2. The protein levels of IRF-4 and its downstream genes in BC3 cells with or without LANA knockdown are shown at the right panel.

To determine if IRF-4 directly interacts with LANA, we performed GST pull-down assays by using a series of *in vitro*-translated truncated mutants of LANA plus the full length molecule incubated with bacterially expressed GST-IRF-4 or GST alone protein. The results showed that the C-terminal domain (945–1162) of LANA strongly interacted with IRF-4 (Figure 6A). Similarly, using *in vitro*-translated IRF-4 co-incubated with bacterially expressed GST-LANA N (1–340) and C (945–1162), the results of GST pull-down assays confirmed that the C-terminus of LANA directly binds with IRF-4 (Figure 6B). To define which domain of IRF-4 is responsible for interacting with LANA, we generated four truncated mutants of IRF-4 with FLAG tag residues (1–271, 145–451, 246–451 and 1–135) and performed similar GST pull-down assays again by using GST-LANA C incubated with *in vitro*-translated truncated mutants of IRF-4. The results showed that IRF-4 bound with LANA through its DNA-binding domain (residues 1–135) at the N-terminus (Figure 6C). The results which showed that in-vitro translated IRF-4 treated with DNase I still bound to LANA (Figure 6B), excluded the possibility that IRF-4 and LANA interaction was dependent on the DNA sequence. To confirm that these two molecules do associate in cells, we performed co-immunoprecipitation assays to determine if LANA forms a complex with IRF-4 in KSHV negative (DG75) and positive (BC3) B-lymphoma cell lines. The results of the co-IP assays from DG75 cells with exogenous LANA and IRF-4 co-expression, showed a complex of IRF-4 with LANA (Figure 7A, lane 6). Consistent with these results, the co-IP studies from BC3 cells showed that endogenous IRF-4 was dramatically immunoprecipitated with anti-LANA antibody, but not using a control mouse serum (Figure 7B, top and left panels). The reverse immunoprecipitation using anti-IRF-4 antibody further confirmed that LANA does form a complex with IRF-4 in KSHV infected cells (Figure 7B, top and right panels). This supports the data above that these two proteins can associate in the same molecular complex.

### LANA knockdown enhances DNA-binding affinity of IRF-4 with CIITA promoter

Since LANA bound to the DNA-binding domain of IRF-4, we wanted to determine if LANA-mediated inhibition of IRF-4 transcriptional activity on CIITA promoter is due to the interaction of LANA with IRF-4. Therefore, we performed chromatin immunoprecipitation assays with exogenous IRF-4 alone or IRF-4 and LANA in the presence and absence of pIII and pIV promoter DNA element. As shown in the Figure 7C, LANA dramatically reduced the ability of IRF-4 to bind to the DNA element of pIII or pIV promoter. To further confirm the consequence of LANA blocking DNA-binding ability of IRF-4 and argue for the possibility that IRF-4 can directly bind to the DNA probe, we also performed reporter assays by co-expressing exogenous IRF-4 with different truncated mutants of LANA N- and C-terminus in the presence of pIII or pIV promoter-driven luciferase reporters in DG75 cells. The results showed that LANA C-terminus instead of N-terminus significantly diminished IRF-4-mediated transcriptional activity of both the pIII and pIV promoters (Figure 7D). Consistent with the results of chromatin immunoprecipitation assays using antibodies against endogenous IRF-4, we observed that IRF-4 had a much stronger affinity when compared to the non-specific IgG control in binding to CIITA pIII promoter once LANA was knocked down in BC3 cells (Figure 7E). In strong support, the levels of well-known IRF-4 downstream targets including c-Myc, CDK6 and Cyclin B1 were also enhanced (Figure 7E). Taken together, these results suggest that LANA can reduce CIITA transcript levels through targeting the binding of IRF-4 to pIII and pIV promoters.

## Discussion

**Figure 8:**
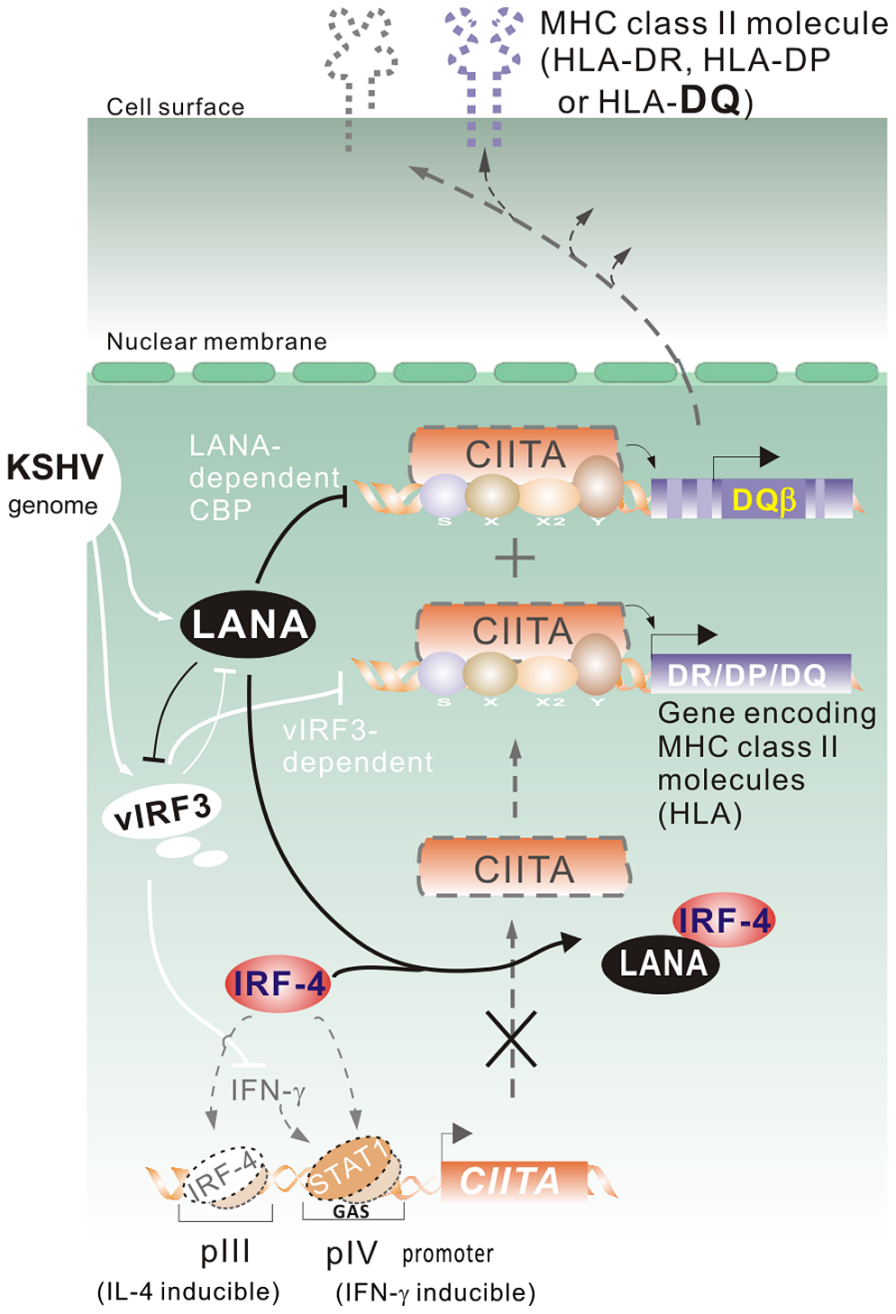
A model depicting the role of LANA on MHC II expression and presentation. In the KSHV-infected cells, the major latent antigen LANA interacts with IRF-4 and reduces the DNA-binding ability of IRF-4 on the IL-4-inducible pIII and IFN-γ-inducible pIV promoter of CIITA, which inhibits the transcriptional activity of CIITA on MHC class II promoter. Meanwhile, associated with this is LANA-mediated inhibition of MHC class II expression and presentation (particularly HLA-DQβ) and other viral genes (like vIRF3) expression in terms of cooperative regulation of host MHC II molecules during viral pathogenesis.

Latency is a phase of the viral life cycle where expressions of encoded proteins susceptible to immune recognition are significantly reduced. During KSHV latency, it has been widely demonstrated that LANA is one of the key latent antigens responsible for KSHV episome maintenance and pathogenesis [50]. In the present study, we further characterize another function that LANA evolves an elaborate mechanism to modulate host immune response through targeting IRF-4-mediated transcription of CIITA. The interaction of LANA with IRF-4 blocks the ability of IRF-4 bound to CIITA promoters, which results in the shut-off of all of CIITA-mediated MHC II molecule expression (HLA-DR, DP and DQ). In contrast, suppression of LANA only led to a specific MHC II namely HLA-DQβ expression and presentation instead of HLA-DQα, DR or DP, as well as the evidence of vIRF3 inhibition further enhanced the HLA-DQβ expression caused by LANA suppression, indicating that LANA is directly involved with the deregulation of CD4^+^ T cells immune response against viral infection through cooperation with other viral antigen like vIRF3. In addition, the inhibition of CBP which results in blocking of LANA-mediated suppression of HLA-DQβ expression indicated that LANA can play a global role on deregulation of MHCII in a CIITA dependent and independent manner (Figure 8).

It has been demonstrated that KSHV contains a cluster of open reading frame encoding proteins with homology to the cellular IRF family [50], and that IFN-γ is transcribed in most KSHV positive PEL cells [38]. Different from these studies, KSHV encodes vIRF-3 or upregulates cellular SOCS3 expression, which were reported to inhibit MHC II expression and presentation through blocking of IFN-γ-STAT1 signaling [38], [51]. In this study, we showed that LANA can directly regulate the transcriptional activity of cellular IRF-4 on the CIITA promoter independent of IFN-γ or IL-4 signaling (we have previously found that LANA blocks IL-4 induced STAT6 signaling pathway [52]). Interestingly, in addition to IRF-4 constitutively activating the pIII promoter as described previously [19], we have now shown that activation of the pIV promoter by IRF-4 requires an intact STAT1-binding site. Furthermore, LANA is able to efficiently block the transcriptional activity of IRF-4 by directly interacting with the DNA-binding domain of IRF-4 instead of the PU.1-binding domain. In contrast, we did not observe any effect of LANA on the pI and pII promoters of CIITA (data not shown). This may explain why LANA can inhibit CIITA through targeting IRF-4 instead of through IFN-γ or IL-4 signaling. Together with the recent report showing that vIRF3 not only modulates MHC II antigen presentation through both CIITA dependent and independent pathway, overexpression of vIRF3 also inhibits endogenous LANA expression [53]. Our studies showing that LANA regulates MHC II in both CIITA dependent and independent manner, as well as the fact that LANA inhibits vIRF3 transcripts, further suggest that LANA and vIRF3 tightly collaborate to control MHC II and CIITA pathway.

It was previously reported that KSHV encoded IRF homologues show some homology to cellular IRF-4, particularly vIRF-3 [54], [55]. To address if anti-IRF-4 antibody recognizes both vIRF-3 and vIRF-4, we performed immunbolotting assay of whole cell lysate from PEL cells by using anti-IRF-4 antibody. The results showed that only a ∼45 kDa protein band of IRF-4 appeared instead of a ∼73 kDa vIRF-3 or a ∼130 kDa vIRF-4 signal which excludes the possibility of cross recognition by the anti-IRF-4 antibody. Furthermore, consistent with the previous report [48], we also found that IRF-4 is constitutively expressed and has clearly different protein modified pattern in the most of PEL cells. In the B-cell context, PU.1 is required for IRF-4 to strongly transactivate downstream target genes. It is intriguing that a complete abrogation of PU.1 expression in PEL cells could be due to LANA deregulation. However, further investigation of IRF-4 downstream gene promoters (i.e. c-Myc, CDK6 and Cyclin B1) will be needed to address this hypothesis.

In addition, the fact that inhibition of CIITA and partial MHC II molecules (other MHCII could be impaired by vIRF-3 expression) in PBMC upon KSHV primary infection is in a LANA-dependent manner, supports the notion that both vIRF-3 and LANA participate in deregulation of MHC II molecules through different targets. The fact that LANA knockdown restores only HLA-DQβ instead of other MHCII molecules, further confirms the conclusion that LANA can precisely regulate specific MHC II molecules not just CIITA expression. However, further investigation is required to address how LANA differentially regulates each HLA molecule independent of CIITA expression. It has been previously demonstrated that CIITA activates MHC II transcription through interactions with the ubiquitously expressed MHC II promoter binding proteins RFX, CREB and NF-Y [56]. In light of the fact that LANA can inhibit CREB *trans*activation by direct interaction or via indirect interaction with CREB-binding protein [57], it is possible that LANA may also influence more cellular genes that are involved in MHC II particularly the HLA-DQβ pathway to intervene in recognition of infected cells by the immune system. Our discovery that knockdown of CBP disrupts the up-regulation of HLA-DQβ by LANA, further confirms that CBP is one of the transcriptional factors targeted by LANA to inhibit MHC II expression. Notably, LANA knockdown did not restore all MHC II molecules except for HLA-DQβ, indicating that there are different subtle regulations of each MHC II molecule in addition to the master regulator CIITA, and that LANA may mainly target the CREB/CBP-associated HLA-DQβ. Due to the complexity of the DQ subtypes (at least DQ1, 2, 3 to 9) in human HLA which has different affinity with specific peptide, it was not surprising for us to observe that LANA knockdown do not enhance specific DQ7 or DQ5 (data not shown) T-cell recognition with particular LANA-derived peptides loading *in vitro*. It is also exclusive that the other KSHV encoded antigens including RTA, responsible for lytic reactivation when LANA is knocked down may be involved in deregulation of MHC II expression. We did not observe any significant change of the same promoter latent antigen vCyclin and vFLIP transcripts.

In summary, the present study has identified a novel role for LANA in down-regulation of CIITA expression through a critical IRF-4 binding site within both CIITA pIII and pIV promoters. By interaction with IRF-4, LANA acts as a viral inhibitor of the MHC class II pathway, negatively regulating CIITA transcription, and thus inhibiting the expression and presentation of MHC II molecules. This further supports an increasing body of evidence which shows that viruses can escape detection of CD4^+^ T cells by inhibiting induction of MHC class II gene expressions by IFN-γ signal transduction or CIITA expression. This property may be important for LANA to contribute to KSHV latency in APCs like endothelial and B cells, and provides a significant strategy for circumvention of the immune response for survival of KSHV.

## Materials and Methods

### Ethics statement

De-identified Human peripheral blood mononuclear cells (PBMCs) were obtained from the University of Pennsylvania CFAR Immunology Core. The Core maintains an IRB approved protocol in which Declaration of Helsinki protocols were followed and each donor gave written, informed consent.

### Plasmids, antibodies and cell lines

DNA constructs expressing LANA full length in the pA3M vector were described previously [58]. GST-LANA N (1–340aa) and C (945–1162aa) in pGEX-2TK were described previously [30]. IRF-4-FLAG and GST-IRF-4 were individually generated by PCR amplicon from pOTB7-IRF-4 (Invitrogen) and inserted into pA3F and pGEX-2TK vector. The truncated mutants of IRF-4 1–135, IRF-4 1–271, IRF-4 145–451 and IRF-4 246–451 with FLAG tag were obtained using the PCR amplicon with BamHI/EcoRV digestion inserted into pA3F vector, respectively. The pIII, pIV, pIV(mGAS) and pIV(mBIimp1) CIITA promoter-driven luciferase reporter constructs were provided by Janet Piskurich (Mercer University School of Medicine) [49]. An HLA-DR plasmid was provided by Richard Longnecker from Northwestern University (Chicago, IL). The monoclonal antibody anti-myc (9E10) was prepared from hybridoma cultures. Mouse monoclonal antibody against HLA-DRα (G-7) or CIITA (7-1H) and rabbit polyclonal antibody against IRF-4 (H-140) were purchased from Santa Cruz Biotechnology, Inc. (Santa Cruz, CA). Mouse monoclonal antibody against Cyclin B1 (V152, Cell Singaling) and FLAG epitope (M2, Sigma) was used.

The KSHV-positive (BC3 and JSC1), KSHV-negative (BJAB, DG75 and Ramos) B lymphoma cell lines were cultured in RPMI 1640 medium supplemented with 7% fetal bovine serum, 2 mM L-glutamine, and penicillin-streptomycin (5 U/ml and 5 µg/ml, respectively). HEK-293 cells were cultured in Dulbecco's modified Eagle's medium (DMEM) supplemented with 5% fetal bovine serum, 2 mM L-glutamine, and penicillin-streptomycin (5 U/ml and 5 µg/ml, respectively). All cell lines were grown at 37°C in a humidified environment supplemented with 5% CO_2_.

### Immunoprecipitation and immunoblotting

Transfected cells were harvested, washed with ice-cold PBS, and lysed in ice-cold RIPA buffer [10 mM Tris-HCl (pH 7.5). 1% Nonidet P-40 [NP-40], 150 mM NaCl, 2 mM EDTA with protease inhibitors]. Lysates were subjected to immunoprecipitation and immunoblotting or directly immunoblotting as described previously [43] .

### Luciferase reporter assay

Reporter assay was essentially performed as described previously [59]. Briefly, at 48 h post-transfection, the cells transiently transfected with the combined plasmids as indicated were harvested and subsequently washed once with PBS (Invitrogen), followed by lysis with 200 µl of reporter lysis buffer (Promega, Madison, Wisconsin, United States). A 40-µg total protein of the lysate was mixed with 100 µl of luciferase assay reagent. Luminescence was measured by the Opticomp Luminometer (MGM Instruments, Hamden, Connecticut, United States) for 10 s. The differences in the amounts of total DNA were normalized with vector control to reach the same amount of total transfected DNA. The luciferase activity was normalized with co-transfected β-galactosidase activity, and presented as fold activation or unit value relative to the reporter construct alone. The results represent experiments performed in duplicate.

### Purification of GST fusion proteins and GST pull-down assays

GST and each GST fusion protein GST-IRF-4, GST-LANA-N and GST-LANA-C were expressed and purified from Escherichia coli BL21 (DE3) as described previously [60]. For GST pull-down assays *in vitro*, GST fusion proteins were incubated with ^35^S-labeled *in vitro*-translated protein in binding buffer (1× PBS, 0.1% NP-40, 0.5 mM DTT, 10% glycerol, supplemented with protease inhibitors) and rotated for overnight at 4°C. After washing three times with binding buffer, proteins were eluted in sample buffer and resolved by SDS-PAGE and then autoradiographed and scanned by a PhosphoImager (Amersham Biosciences). *In vitro* translation was performed with the T7-TNT Quick Coupled transcription-translation system (Promega Inc., Madison, WI) according to the manufacturer's instructions.

### Chromatin immunoprecipitation assay

HEK-293 cells (10×10^6^) transfected IRF-4-FLAG or IRF-4-FLAG with LANA-C-myc in the presence of pIV-WT, pIV mGAS or vector were individually subjected to chromatin immunoprecipitation (ChIP) analysis as previously described [60]. Briefly, Cells were cross-linked with 1.1% (v/v) formaldehyde, washed with PBS, incubated with Buffer A [10 mM Tris-HCl (pH 8.0), 10 mM EDTA, 0.5 mM EGTA, and 0.25% (v/v) Triton X-100], Buffer B [10 mM Tris-HCl (pH 8.0), 1 mM EDTA, 0.5 mM EGTA, and 200 mM NaCl] and finally sonicated Buffer C [10 mM Tris-HCl (pH 8.0), 1 mM EDTA, 0.5 mM EGTA, 1% (w/v) SDS plus 1 mM PMSF, 1 µg/ml aprotinin, leupeptin, and pepstatin] to generate an average fragment size of 300–500 bp. 20% of solubilized chromatin extracts were saved for input followed with cross-link reverse step, and the remaining were clarified by centrifugation and diluted to 6 OD_260_ U/ml in IP buffer [140 mM NaCl, 1% (w/v) Triton X-100, 0.1% (w/v) sodium deoxycholate, 1 mM PMSF, 100 µg/ml salmon sperm DNA, and 100 µg/ml BSA]; preincubated with 50% (v/v) Protein A-agarose (Invitrogen Life Technologies, Camarillo, CA) with normal mouse/rabbit sera (Invitrogen, Carlsbad, CA); reconstituted in PBS, and washed several times in IP buffer. Aliquots (600 µl) were incubated with 20 µg of each specific antibody for overnight at 4°C. Immune complexes were separated into bound and unbound complexes with Protein A-agarose and cross-links were reversed by treatment at 65°C overnight. After treatment with RNase A and proteinase K, samples were extracted once with phenol/chloroform, and the DNA was precipitated with 2 volumes of ethanol. Precipitated DNA was pelleted, washed once with 70% ethanol, dried, and resuspended for quantitative PCR by using Ampicilin primers (forward: 5′-CATCTTACGGATGGCATGAC-3′, reverse: 5′-CAACGATCAAGGCG AGTTAC-3′).

### KSHV virion purification and primary infection

The 293/Bac36 (GFP-KSHV) cells were subjected to induction by TPA and Sodium butyrate for KSHV virion production. The purified viral suspension was performed to primary infection by using 10^7^ PBMCs (T/B cells ratio is about 7.2) followed by infection protocol as previously described [42], [43], [61].

### Quantitative PCR

Total RNA from cells was extracted using Trizol reagent and cDNA was made with a Superscript II reverse transcription kit (Invitrogen, Inc., Carlsbad, CA). The primers for real-time PCR were as followings: for pIII: 5′-CCTGGCTCCACGCCCTG-3′ and 5′-GAACTGGTCGCAGTTGATG-3′; for pIV: 5′-GAGCTGGCGGCAGGGAG-3′ and 5′-GAACTGGTCGCAGTTGATG-3′; for HLA-DRα: 5′-TTTGAGGCTCAAGGTGCATTG-3′ and 5′-TGGAGGTACATTGGTGA TCGG-3′; for HLA-DRβ: 5′-CCGAGTACTGGAACAGCCAGAA-3′ and 5′-TGCAC TGTGAAGCTCTCACCAA-3′; for HLA-DPα: 5′-GCCCTGAAGACAGAATGTTCCA-3′ and 5′-GCGGCATAAGTTGACACATGG-3′; for HLA-DPβ: 5′-ACAGTCTGATTCTG CCCGGAGT-3′ and 5′-CTTGCTCCTCCTGTGCATGAAG-3′; for HLA-DQα: 5′-ATCAT CCAAGGCCTGCGTT-3′ and 5′- TCTTCTGCTCCTGTAGATGGCG-3′; for HLA-DQβ: 5′-GCAGAGACTCTCCCGAGGATTT-3′ and 5′- CGCACGATCTCTTCTCGGTTAT-3′; for LANA: 5′-CATACGAACTCCAGGTCTGTG-3′ and 5′-GGTGGAAGAGCC CATAATCT-3′; for NF-κB: 5′-GTTTCGGCGGTGGTAGTGGT-3′ and 5′-GGGCT CCTGATCTTGCTCTG-3′; for vIRF-3: 5′-CAGTGTACGGCTCGAGAA GCA-3′ and 5′-TCCTCCTCACTGGAATCCGAGTT-3′[38]; for CBP: 5′-AATCTTTAAA CCAGAGG AGTTACGC-3′ and 5′-ATTATAGAGCCAGGCATTGTTGAAC-3′; and for GAPDH: 5′-CTCCTCTGACTTCAACAGCG-3′ and 5′-GCCAAATTCGTTGTCATACCAG-3′. The cDNA was amplified by using 10 µl of Master Mix from the DyNAmo SYBR green quantitative real-time PCR kit (MJ Research, Inc.), 1 µM of each primer, and 2 µl of the cDNA product in a 20-µl total volume. Thirty cycles of 1 min at 94°C, 30 s at 55°C, and 40 s at 72°C were followed by 10 min at 72°C in an Step-One thermocycler (Applied Bio Inc.). A melting curve analysis was performed to verify the specificities of the amplified products. The values for the relative levels of change were calculated by the “delta delta threshold cycle” (ΔΔC_T_) method and each sample were tested in triplicates.

### RNA interference

Small hairpin RNAs (shRNA) complementary to the GCTAGGCCACAACACATCT fragment of LANA as described previously [58], AAGGCCATTTGTGGGTGAAAA fragment of vIRF3 [62], GCTTCCCACACTATGGA TTTC fragment of NF-κB, or GGGAAATGTCTGAATCTTTCC fragment of CBP was individually cloned into pGIPz vector according to the manufacturer's instructions (Open Biosystem, Inc, Huntsville, AL) to generate shLANA, shvIRF3, shNF-κB and shCBP constructs. pGIPz vector with luciferase target sequence (shCtrl) was used as a control. Fifteen million PEL cells were transduced by lentivirus packaging specific shRNA or directly transfected by electroporation (220v, 975 µF, 0.4-cm-gap cuvette) with 20 µg of specific shRNA as indicated.

### Northern blot

Total RNA of BJAB and DG75 cells transduced with lentivirus carrying LANA-YFP or vector alone was extracted by using Trizol reagent (Invitrogen, Inc., Carlsbad, CA). 10 µg RNA/well were electrophoretically separated on an agarose gel and transferred to nylon membrane (Hybond-N Amersham Corp. Arlington Heights, IL) by the pressure transfer method. A 1.3 Kb specific for HLA-DR α chain from HLA-DR plasmid was isolated and labeled with [^32^P]dCTP by random-primed synthesis. Reaction products were purified from unincorporated isotope via a Mini Spin G-50 column (Worthington Biochemical Corp. Freehold, NJ), melted, hybridized and detected as previously described [63]. After hybridization overnight at 42°C, the final wash was carried out at 56°C with 0.2×SSC and 0.1% SDS for 30 min. Autoradiography was performed by a PhosphoImager (Amersham Biosciences) with stored at -80°C for 4–8 hr.

### FACS analysis

One million of PEL cells (JSC1 or BC3) were harvested 2 days after transfection with shRNA expression plasmid, respectively. The cells were washed once with phosphate-buffered saline (PBS) and once with FACS buffer (2% fetal calf serum [FBS] and 0.05% sodium azide in PBS) and then incubated with phycoerythrin (PE)-coupled primary antibody against human HLA-DR (1∶5 dilution, clone# TU36, BD Biosciences), DP (1∶100 dilution, clone# B7/21, Abcam) and DQ (1∶5 dilution, clone# HLA-DQ1, BioLegend) in FACS buffer for 20 min at room temperature. After 3 washing steps with FACS buffer, the cells were fixed by 2% PFA and analyzed on a FACSCalibur system (BD Biosciences, San Joe, CA) and the results were analyzed using the FlowJo software (Tree star, Ashland, OR).

### T cell recognition assays

Transduced cell lines were sensitized with ten-fold dilutions of peptide ranging from 10^−5^ M to 10^−10^ M for one hour at 37°C, before being washed five times. These target cells were plated out in v-bottom 96 well plates at 50000 cells per well in RPMI-1640 with 10% FCS. Effector cells were CD4+ T cell clones established and grown as described in previous studies [41], [64]. These clones were specific for the peptides GSPTVFTSGLPAFVS or PAFVSSPTLPVAPIP derived from LANA and presented by HLA-DQ7. Effector cells were plated onto the target cells to give 5000 CD4+ T cells per well. As controls, T cells were incubated in the absence of target cells to measure spontaneous activity, or incubated with EBV transformed B cells either sensitized with 10^−6^ M of the cognate peptide or an equivalent dilution of the peptide solvent dimethyl sulphoxide or an HLA mismatched B cell. Cultures were incubated for 18 hours after which supernatants were harvested and assayed for the presence of IFN-γ by ELISA (Endogen) and concentrations determined by comparing to known concentrations of recombinant IFN-γ standards.
